# Frost Induces Respiration and Accelerates Carbon Depletion in Trees

**DOI:** 10.1371/journal.pone.0144124

**Published:** 2015-12-02

**Authors:** Or Sperling, J. Mason Earles, Francesca Secchi, Jessie Godfrey, Maciej A. Zwieniecki

**Affiliations:** 1 Department of Plant Sciences, PES #2316, One Shields Avenue, University of California Davis, Davis, CA 95616, United States of America; 2 Ecology Graduate Group, University of California Davis, Davis, CA 95616, United States of America; 3 Department of Agricultural, Forest and Food Sciences (DISAFA), University of Torino, Via 15 Leonardo da Vinci 44, 10095 Grugliasco, TO, Italy; University of California Davis, UNITED STATES

## Abstract

Cellular respiration depletes stored carbohydrates during extended periods of limited photosynthesis, e.g. winter dormancy or drought. As respiration rate is largely a function of temperature, the thermal conditions during such periods may affect non-structural carbohydrate (NSC) availability and, ultimately, recovery. Here, we surveyed stem responses to temperature changes in 15 woody species. For two species with divergent respirational response to frost, *P*. *integerrima* and *P*. *trichocarpa*, we also examined corresponding changes in NSC levels. Finally, we simulated respiration-induced NSC depletion using historical temperature data for the western US. We report a novel finding that tree stems significantly increase respiration in response to near freezing temperatures. We observed this excess respiration in 13 of 15 species, deviating 10% to 170% over values predicted by the Arrhenius equation. Excess respiration persisted at temperatures above 0°C during warming and reoccurred over multiple frost-warming cycles. A large adjustment of NSCs accompanied excess respiration in *P*. *integerrima*, whereas *P*. *trichocarpa* neither excessively respired nor adjusted NSCs. Over the course of the years included in our model, frost-induced respiration accelerated stem NSC consumption by 8.4 mg (glucose eq.) cm^-3^ yr^-1^ on average in the western US, a level of depletion that may continue to significantly affect spring NSC availability. This novel finding revises the current paradigm of low temperature respiration kinetics.

## Introduction

Plant vitality may be compromised if demand for non-structural carbohydrates (NSCs) exceeds supply over prolonged periods of time. Such an NSC imbalance may result in the reduction of growth, loss of reproductive capacity, or delayed recovery from stress. Under extreme circumstances, excessive NSC depletion may kill the plants [1–3]. During periods of limited photosynthesis, like winter dormancy or drought stress, trees depend solely on stored NSCs to maintain basic metabolic functions, produce defensive compounds, and retain cell turgor [4,5]. With amassing threats to plants due to climate change, knowing how environmental factors affect NSC demand is critical for agricultural and forest management, modeling climate impacts, and better understanding plant dormancy biology.

**Fig 1:**
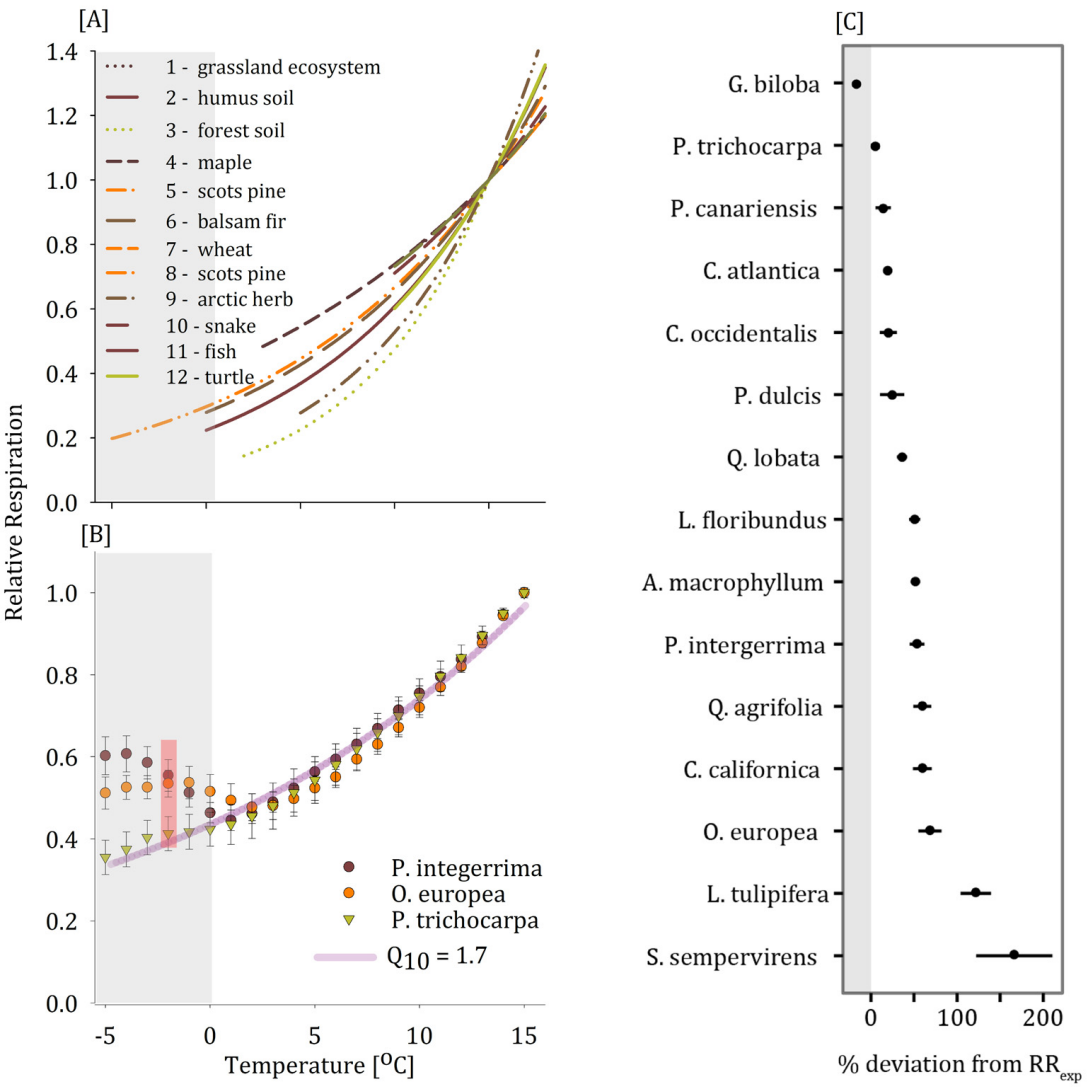
(A) The effect of temperature on relative respiration (the fraction of respiration relative to 15°C) of common species including animals, microbes, and plants follows the Arrhenius exponential derivative (Q_10_) that ranges between 1.5 and 2.7 (see S1 Table for references). (B) The effect of temperature on relative stem respiration (t_0_ = 15°C and Rs_0_ = 390 μmol CO_2_ m^-3^ s^-1^) for *P*. *integerrima*, *P*. *trichocarpa*, and *O*. *europea* followed a single exponential trend between 1°C and 15°C (Q_10_ of 1.7 –pink line). A significant divergence from the predicted exponential respiration drop was observed for *P*. *integerrima* and *O*. *europea* near 0°C (red bar indicates differences at -2°C in an unpaired two-way t-test, p<0.005, df = 14). *P*. *trichocarpa* shows no significant divergence from the predicted exponential curve at low temperatures. (C) The percent deviation in respiration at -2°C for 15 tree species (mean ± SE of 5 replicates).

Cellular respiration is the primary energy generator for all cellular activities and, therefore, constitutes a major sink for NSCs, especially when growth is limited. As an enzymatic process, respirational activity is intimately linked to the environment via temperature. Thus, like many other biological processes, its thermal kinetics can be represented by the Arrhenius equation [6] and the thermal coefficient, $Q_{10}={\left(\frac{R_{2}}{R_{1}}\right)}^{\left[10/\left(T_{2}-T_{1}\right)\right]}$, where R_1_ and R_2_ are respiration rates, and T_1_ and T_2_ are the temperatures at which respiration is measured. Respirational Q_10_ varies between 1.3 and 2.7 for many biological systems, e.g. single enzymes in the respiratory pathway [7], mitochondria [8], soil microbes [9], various plants [10–12], animals [13], fungi [9], and whole ecosystems [14] (Fig 1A). In each of these systems, Q_10_ is considered constant across the wide range of inhabitable temperatures. However, plants are known to have a variable Q_10_ [11], which is mainly associated with high temperatures and attributed to substrate availability, enzyme denaturation [15], and/or temperature acclimation [16].

Despite prior knowledge that trees may actively acclimate to cold and prepare for frost near 0°C [17,18], little is known about how temperature affects respiratory activity at or near freezing temperatures. During the onset of freezing, plants need energy to stimulate frost protection via soluble sugar accumulation [19] and/or to produce anti-freeze proteins [20]. This is in addition to the demands of routine metabolic activities. Interestingly, the need for frost protection coincides with decreasing metabolic rates, raising the question of how frost protection is metabolically fueled. Further, in many environments freezing also coincides with dormancy and drought in trees, i.e. periods of limited photosynthesis and increased reliance on stored NSCs. Despite its potential importance, the impact of temperature on stored NSCs during physiological dormancy has not been previously studied. Thus, we examined the: 1) dynamics of respiration at near-zero temperatures in woody species, 2) potential effects of frost-induced respiration on soluble and insoluble NSC levels, and 3) spatio-temporal patterns of modeled NSC depletion across the western US based on historical temperature patterns.

## Materials and Methods

### Plant materials

We collected *Acer macrophyllum* Pursh (big leaf maple), *Carpenteria californica* Torr. (bush anemone), *Cedrus atlantica* (Endl.) Manetti ex Carrière (atlas cedar), *Cercis occidentalis* Torr. ex Gray (western redbud), *Ginkgo biloba* L. (ginkgo), *Liriodendron tulipifera* L. (tulip tree), *Lyonothamnus floribundus* A. Gray (Catalina ironwood), *Olea europaea* L. (olive), *Pinus canariensis* C. Sm. (Canary Island pine), *Pistachia integerrima* L. hybrid (pistachio), *Populus trichocarpa* Torr. & A. Gray (poplar), *Prunus dulcis* (Mill.) D. A. Webb (almond), *Quercus agrifolia* Née (coast live oak), *Quercus lobata* Née (valley oak), and *Sequoia sempervirens* (D. Don) Endl. (coastal redwood) branches from trees in the experimental farms and arboretum of the University of California, Davis (38.54°N, 121.80°W) during fall of 2014 (at ca. 0900 AM). We collected mature branches (second order with thick dark bark) with diameter of ca. 10 mm and length > 50 cm from 5 different trees (one branch per tree). We cut branches segments with no leaves and transferred them in a moist plastic bag to the lab. In the lab we re-cut the branches under water, at least 5 cm away from the original cut, and sealed the ends with parafilm prior to subsequent measurements.

### Response of stem respiration to temperature

We gradually cooled *P*. *integerrima*, *O*. *europea*, and *P*. *trichocarpa* branches from 15°C to -5°C in 4 hours (5°C h^-1^), maintained them at -5°C for 3 hours, and heated them to 15°C in 2 hours (10°C h^-1^). This protocol roughly characterizes the early winter frost events we measured in the field (Central Valley, CA) where air cooled gradually after sundown, reached minimum temperature between 0200 and predawn, and then heated quickly as the sun came up. We kept air flow in the cooling chamber constant at 200 μmol s^-1^, the reference CO_2_ at 400 ppm, and matched the humidity to ambient conditions. CO_2_ efflux rates were continuously monitored (Licor 6400, LiCor Inc., Lincoln, NB, USA) and respiration was computed according to *R* = *J*_*CO*2_ × *A*_*cuvette*_/*V*_*branch*_; where J_CO2_ is the CO_2_ efflux out of the stem, A_cuvette_ is the surface of the Licor’s leaf chamber (0.0006 m^2^), and V_branch_ is the volume of measured branches (ca. 0.001 m^3^). Relative respiration was computed as *RR* = *R*_*T*_/*R*_15_ were R_T_ is respiration at temperature T and R_15_ is respiration at 15°C (measured for each stem independently). We validated these measurements by comparing our findings to stem respiration of intact *P*. *integerrima* saplings. The saplings were two years old, grown in 10 L pots, and fully hydrated (stem water potential of -0.25 MPa). We transferred saplings to the lab and after they had acclimated for 30 minutes we covered them with an impermeable bag to maintain high internal relative humidity and avoid further water loss then began measurements. The temperature-controlled chamber was placed around a 15 cm long stem segment (~10 mm in diameter), so that the tree passed through the chamber. In this way we avoided removing saplings from their pots, but were still able to measure the CO_2_ efflux from a temperature-treated segment of the stem. The respiration kinetics of intact and excised stems was indistinguishable (see Fig 1 and S1 Fig).

To enlarge our sample size and the number of species examined, we constructed an additional setup with five sealed falcon tubes in a temperature-controlled chamber. The aluminum chamber was insulated by a 3 cm layer of polystyrene and, thus, the tubes were in the dark. The tubes were connected to a manifold that enabled consecutive respiration measurements of 5 branches (10 cm long) using a gas analyzer (Licor 6262, LiCor Inc., Lincoln, NB, USA). Air flowed constantly through the tubes at 220 μmol s^-1^ to avoid CO_2_ accumulation around the branches between measurements. Respiration was recorded at four temperatures during cooling: 15°C, 10°C, 5°C, and -2°C. Each temperature was applied for 0.5 hours before measurements were taken. We used the respiration values at 15°C, 10°C, and 5°C to fit an exponential curve and determine a species-specific Q_10_ value. Then, we calculated the projected respiration at -2°C and the associated percent deviation from the projected value using the Arrhenius equation [6] at -2°C.

To verify that excess respiration persists on a diurnal time scale and across multiple days, we measured stem respiration of four *P*. *integerrima* saplings that were repeatedly treated for 24 hour periods. Each day we cooled the trees from 22°C to -2°C in 7 hours (2300–0600), heated them to 22°C in 5 hours (0600–1100), and then maintained them at 22°C for the remaining 12 hours (1100–2300). We placed the temperature controlled (and CO_2_ analyzed) chamber on 15 cm long stem segments with the trees intact and applied light (1000 μmol _photons_ m^-2^ s^-1^) between 0600 and 1800 (meeting the ambient photoperiod at the time). Respiration was continuously monitored by the Licor 6262. We repeated this cooling/heating cycle in each tree for three consecutive days. We monitored stem temperature every 10 seconds and did not observe exotherms, therefore excluding the possibility of stem freezing.

### Determination of non-structural carbohydrates

We measured soluble carbohydrates in the wood parenchyma cells (i.e. excluding bark and phloem) according to Leyva *et al*. [21]. In short, 1 mL of deionized water was added to 50 mg of dried tissue, vortexed, heated to 72°C for 15 min, and spun at 21000 rcf for 10 min. A 50 μL aliquot of the supernatant was diluted (x25) and mixed with 150 μL of sulfuric acid (98%) and anthrone (0.1%, w/v) solution in a 96-well micro-plate. The precipitated pellet was reserved for later starch analysis. The plate was cooled on ice (<4°C) for 10 minutes, then heated to 100°C for 20 minutes, and finally left to adjust to room temperature for 20 minutes (22°C). We determined the sugar levels as glucose equivalents from the colorimetric reading (Thermo Scientific Multiskan) of absorbance at 620 nm (A_620_) using a predetermined standard curve (0, 0.01, 0.03, 0.1, and 0.3 mg L^-1^ glucose), and multiplied the outcome by a measured average wood density of 0.63 g cm^-3^.

We quantified starch in the remaining pellet using a starch assay kit (STA-20, Sigma-Aldrich) according to a protocol that we modified (JME, FS, and OS). First, we washed the pellet twice in 80% (v/v) ethanol, spun it at 21000 rcf for 10 minutes, and then disposed of the supernatant. The pellet was digested with α-amylase and α-amyloglucosidase at 100°C and 60°C incubations for 5 and 15 minutes, respectively. Finally, we determined the starch level by measuring the amount of glucose released by the glucose oxidase-mediated assay (STA-20, Sigma-Aldrich) according to a colorimetric reading at 540 nm (A_540_) and multiplied the result by wood density resulting in units of mg cm^-3^.

### Estimating recent NSC consumption due to excess respiration

**Fig 2:**
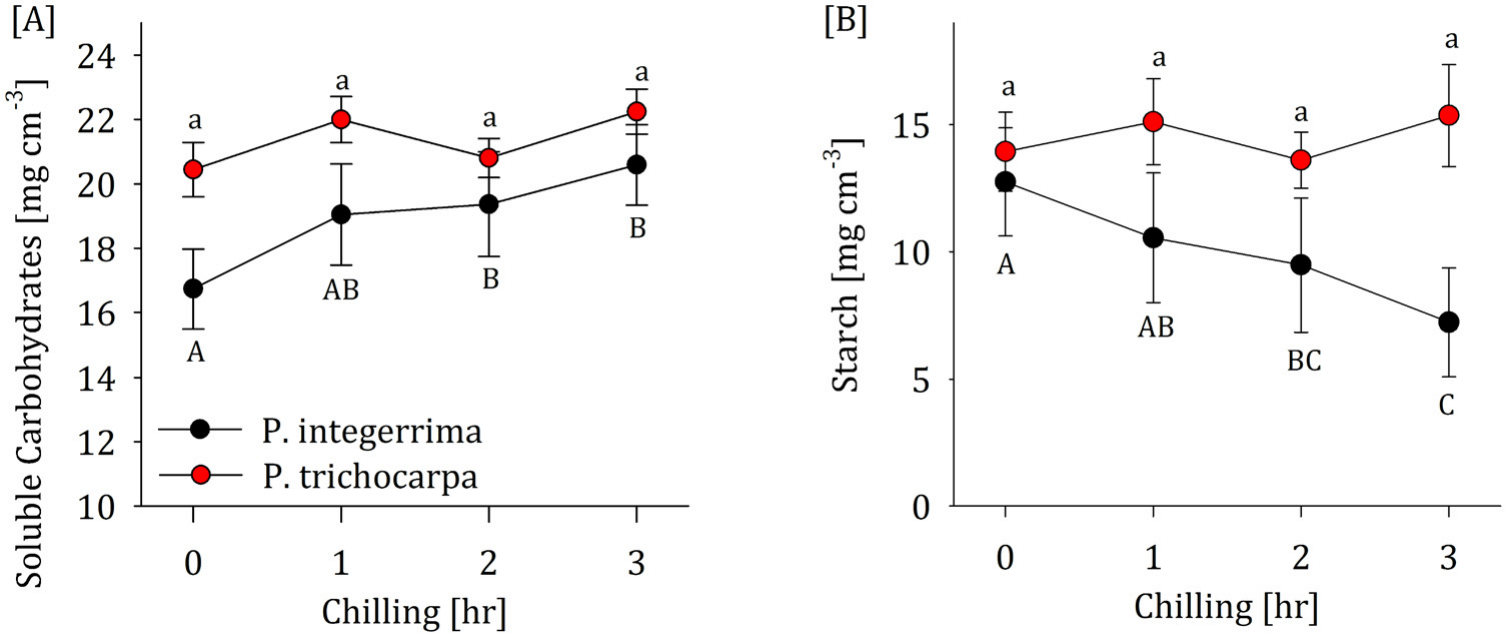
(A) The temperature-respiration relations in *P*. *integerrima* stems during 5 hours of cooling from 15°C to -2°C (blue dots) and 3 hours of heating back to 15°C (orange dots) versus the values projected by the Arrhenius equation (dashed line). Asterisks denote respiration rates which significantly differ from the projected values (paired two-tailed t test, p<0.005, df = 7). (B) The percent deviation from the projected respiration at -2°C during three consecutive days in which statistical analysis showed no significant differences (Repeated measure ANOVA and Tukey HSD, p = 0.05, df = 10).

We estimated NSC consumption due to excess respiration for 2005 to 2013 in the western US using historic 1/8 degree gridded daily maximum and minimum temperature data derived from the coupled general circulation model MIROC5 [22]. Hourly temperature interpolations were performed using the Interpol.T package [23] in R statistical software, which combines linear, parabolic and sinusoidal functions to interpolate between daily minimum and maximum temperatures [24,25]. We estimated respiratory activity in response to hourly temperature data by alternating between two temperature-respiration response functions. Under ‘normal’ circumstances we used the equation R_no_frost_ = 197.6e^0.0457T^, based on the average Q_10_ for all 15 species examined. However, if a frost event (i.e. T < 0°C) occurred in the past 2 hours and T < 15°C and T > -5°C, we used the equation R_frost_ = 245.4e^0.0273T^ derived from observations of *P*. *integerrima* (Fig 2C) which exhibited an intermediary excess respiration response (Fig 1C). Excess respiration was calculated by taking the difference between two scenarios—one in which we assumed the frost-related increase in respiration described above occurs and another in which only R_no_frost_ was used. Based on hourly respiration, we estimated NSC demand, assuming that respiration of 1 mole of glucose releases 6 moles of CO_2_ and that glucose has a molecular mass of 180.2 g mol^-1^. We then summed hourly NSC demand for each year from 2005 to 2013, resulting in mg (glucose eq.) cm^-3^ tissue yr^-1^ for each grid cell.

### Statistical analysis

**Fig 3:**
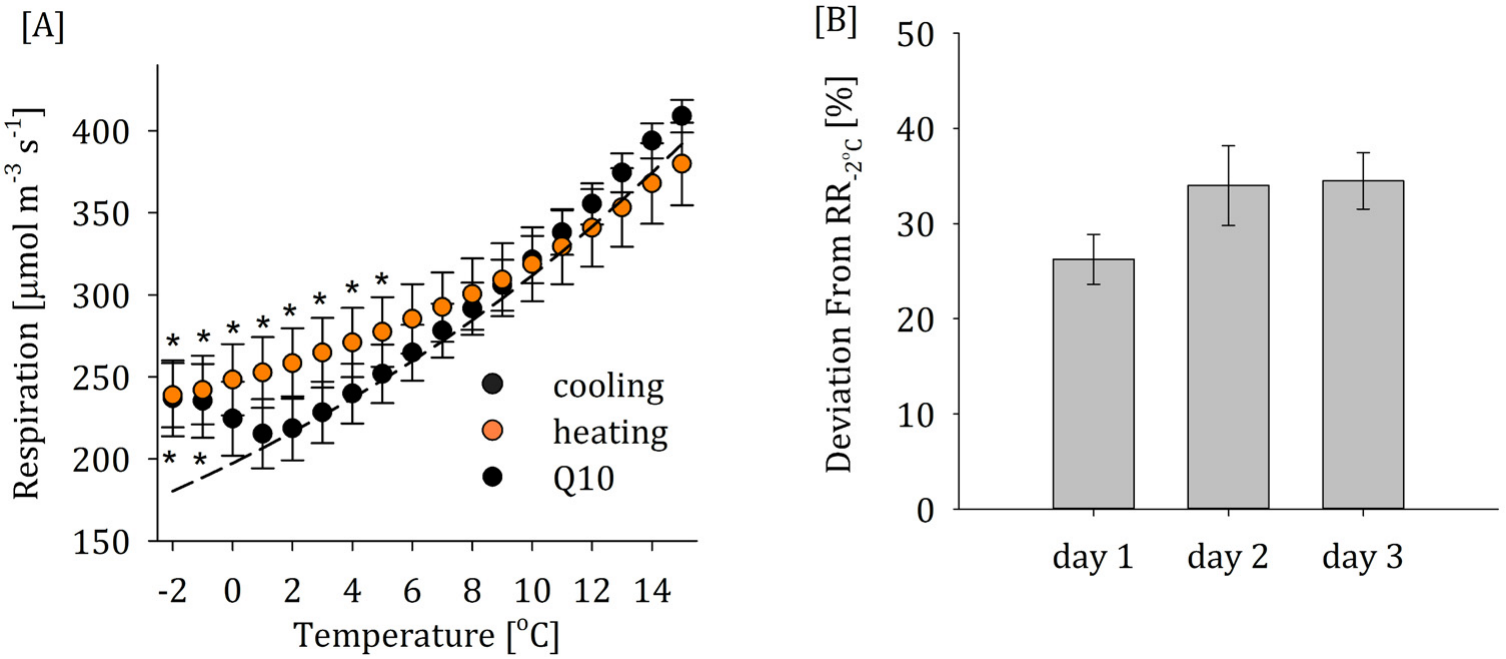
Temporal changes in nonstructural carbohydrate (NSC) concentrations [mg cm^-3^] in stems after 0 (no chilling), 1, 2, and 3 hours of chilling of *P*. *trichocarpa* and *P*. *integerrima* stems (A)–soluble carbohydrates (SC), and (B)–starch. Lower case letters denote significant differences between NSC levels in *P*. *trichocarpa* and upper case letters between NSC levels in *P*. *integerrima* (Repeated measure ANOVA and Tukey HSD, p = 0.05, df = 18).

**Fig 4:**
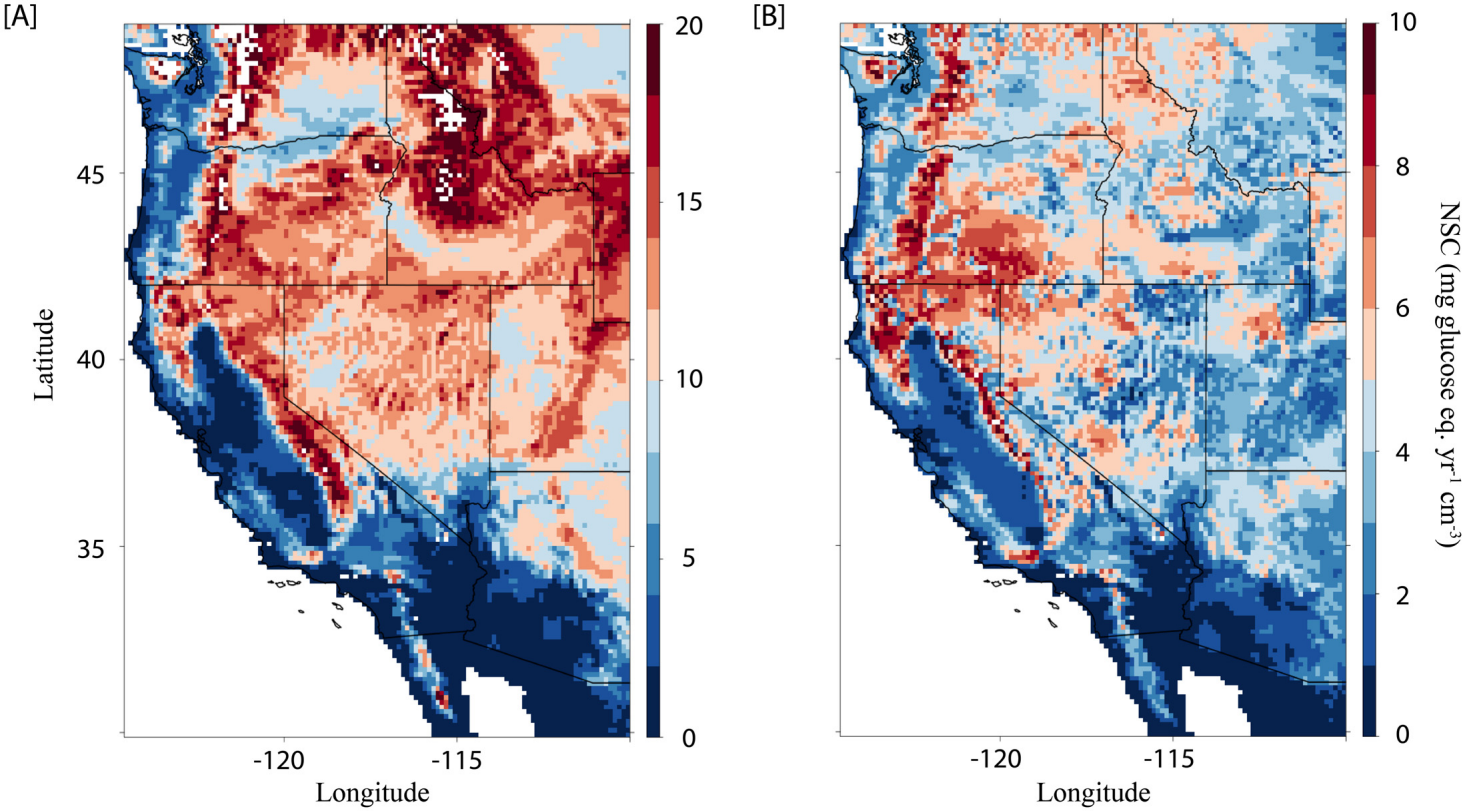
For 2005 to 2013 in the western US, (A) estimated median non-structural carbohydrate (NSC) consumption due to excess respiration and (B) range of variation (min–max) in NSC consumption due to excess respiration.

We used the R (R version 3.2.1) base package to compute statistical differences of means by an unpaired two-way T-test in Fig 1B and paired two-way T-test in Fig 2A (p<0.05). In Figs 2B, 3A and 3B we used a repeated measure ANOVA and Tukey HSD, (p<0.05) to compare the differences in means. For the spatial and temporal temperature interpolations in Fig 4A and 4B we used the ‘Interpol.T’ R package [23] to produce hourly climatic data from local daily minimum and maximum temperatures.

## Results

### Numerous tree species excessively respire near 0°C

Plants, animals, and soil microbes reduce their respiration as temperature drops from 30°C to -8°C (Fig 1A and references therein) and prior to water crystallization. In this study, we measured the respirational activity of branches from 15 tree species. For *P*. *trichocarpa*, *O*. *europea*, and *P*. *integerrima* respiration was measured while slowly cooling branches from 15°C (where respiration averaged 390 ± 20 μmol CO_2_ m^-3^ s^-1^) to -5°C. Unexpectedly, *O*. *europea* and *P*. *integerrima* increased respiration at or near 0°C and maintained elevated respiration for several hours (Fig 1B). In the case of *P*. *integerrima*, the increase in respiration began at temperatures above 0°C and deviated nearly 50% from the expected Q_10_ values when temperature declined to -5°C (Fig 1B). However, *P*. *trichocarpa* branches did not significantly diverge from the expected temperature-respiration curve at any point (Fig 1B). We repeated this measurement at discrete temperatures of 15°C, 10°C, 5°C, and -2°C, for 75 branches total, 5 of each for 15 tree species total (see Materials and Methods). Of these species, 13 increased respiration above expected values at -2°C (Fig 1C), with deviations of up to 170% in the case of *S*. *sempervirens*. Once more, *P*. *trichocarpa* did not deviate above expected respiration at -2°C, and *G*. *biloba* was slightly lower than expected.

### Excess respiration persists at temperatures above 0°C and reoccurs over multiple frost-warming cycles

We examined whether or not excess respiration persists at T > 0°C and repeatedly occurs given multiple frost-warming cycles for *P*. *integerrima*. Following the onset of excess respiration near 0°C, we increased temperatures to 15°C. As temperature increased, respiration remained significantly higher than predicted given the original Q_10_ of 1.6 (Fig 2A). At the same time, warming lowered the Q_10_ to 1.3. When frost-warming episodes were repeated nightly for three subsequent nights, cold-induced respiration deviated by 32% from the projections, with no significant difference between days (Fig 2B).

### NSC depletion accompanies excess respiration

Two species which differ markedly in their respirational response to frost, *P*. *trichocarpa* and *P*. *integerrima*, showed significant differences between the temporal dynamics of their soluble carbohydrate (SC) levels. Initial SC level was higher in *P*. *trichocarpa* compared to *P*. *integerrima—*at 20.5 and 16.8 mg cm^-3^, respectively (Fig 3A). Initial starch levels, on the other hand, were similar and averaged 14 mg cm^-3^ (Fig 3B). Yet during 3 hours of chilling at -4°C, *P*. *integerrima*, which exhibited frost-induced respiration, also increased SC levels in wood parenchyma cells by nearly 25% (4 mg cm^-3^). This increase in SC levels was accompanied by a 45% (6 mg cm^-3^) decrease in starch levels. On the other hand, *P*. *trichocarpa*, which showed no freezing-related excess respiration, did not change cellular SC levels or degrade any starch during chilling. The total loss of 2 mg cm^-3^ in stem NSC (SC + starch) seen in *P*. *integerrima* cannot be attributed to respiration, which accounts for ~0.1 mg cm^-3^ loss of stored carbohydrates integrated over the same period. Hence, some carbohydrates must have been related to irreversible frost-induced processes like cellular damage, cellular repair, or the production of compatible solutes for cellular protection.

### Excess respiration could accelerate NSC depletion in the western US

We developed a spatially-explicit model in which temperature patterns drive non-structural carbohydrate consumption via respiration. We parameterized the model with and without excess respiration to quantify potential effects on annual carbohydrate depletion in trees across the western US. Given temperatures observed from 2005 to 2013, our model estimated that excess respiration depleted trees’ non-structural carbohydrate reserves on average by 8.4 mg (glucose eq.) cm^-3^ yr^-1^ in the western US, with 1^st^ and 99^th^ percentiles below and above 0.0 and 17.7 mg (glucose eq.) cm^-3^ yr^-1^, respectively (Fig 4A). Year-to-year, certain locations exhibit fluctuations in NSC demand due to excess respiration of up 10.6 mg (glucose eq.) cm^-3^ yr^-1^ (Fig 4B), suggesting substantial variability due to site-specific climatic differences.

## Discussion

We show here, for the first time, that numerous tree species (13 out of 15 examined) respond to cooling at near-freezing temperatures by significantly increasing stem respiration rates (Fig 1B and 1C). We attribute this excess respiration to metabolic processes associated with known frost protection mechanisms that result in changes to NSC levels and the production of frost-protection compounds. Knowing that this finding may seem controversial, we thoroughly validated our measurements and concluded that the observed increase in stem respiration rate in response to near 0°C is not an artifact for the following reasons:

- Several tree species did not exhibit a significant increase in respiration near 0°C (Fig 1B and 1C). In fact, we measured a range of responses, from zero to double the respiration predicted by the original Q_10_.
- Stem respiration of both intact potted saplings (*P*. *integerrima*) and excised branch segments exhibited no differences in their cold respiration kinetics (S1 Fig, unpaired two-way T-test, p>0.68, df = 9). They increased respiration in similar proportions and timing, implying that within the experimental time frame, branches served as a reasonable proxy for stem respiration of whole trees.
- The branches were not transpiring and were no longer connected to roots and therefore gas carried in xylem was not the source of excess CO_2_ efflux.
- The gas exchange system had no leaks—CO_2_ measurements of an empty chamber or a chamber with dead *P*. *integerrima* branches (boiled for 30 minutes) exhibited no CO_2_ effluxes.
- Water did not crystalize in the stems. We continuously monitored the temperature (10 second readings and data storage in CR1000, Campbell Scientific, Logan, UT, USA) and none of the branches exhibited an exotherm, precluding ice formation and gas release from freezing water as an explanation for our observations.
- We also measured electrolyte leakage after plant exposure to -5°C to determine if the treatment caused frost and cellular damage. The frost damage index [26] was -1% ± 5% and not significantly different from zero (t-test p>0.29, df = 9) assuring that no frost-related damage occurred.
- The CO_2_ did not come from xylem sap (see Lintunen *et al*., 2014) as gas solubility in sap increases at low temperatures [27–28] even in below 0°C super-cooled water. Furthermore (referring to Lintunen *et al*., 2014), the CO_2_ efflux wasn’t a sudden burst but a continuous release and it didn’t happen in boiled branches perfused with xylem sap that were cooled to temperatures below 0°C.
- The observed spike in respiration is not thermo-regulatory, as observed in flowers which release heat to attract pollinators [29], as our stems continued to cool. Moreover, the energy released during excess respiration is too small to support actual heating.
- The respirational increase repeatedly occurred over multiple frost-warming cycles. We observed respiration for four *P*. *integerrima* saplings over three consecutive nights. The increase in CO_2_ efflux occurred each time. In addition, CO_2_ efflux remained high after plants were re-heated until stems reached 15°C.

Cold-induced metabolism is often related to cold acclimation and frost protection. As intracellular liquid ice crystallization can result in irreversible cell damage [30,31], maintaining the symplast in a liquid state and protecting membrane functionality are key to preserving cellular integrity. This can be achieved by lowering the freezing depression point (i.e. the temperature at which water freezes) [19] or maintaining the symplast in a super-cooled state [18], which requires the accumulation of soluble sugars (Fig 2A) and the production of compatible osmolytes [20]. Two species with distinct differences in their respirational response to frost exhibited corresponding differences in their temporal dynamics of SC (Fig 2A) and starch (Fig 2B) levels. *P*. *integerrima*, which increased respiration during frost events, increased its SC levels in wood parenchyma cells by nearly 25% (4 mg cm^-3^) during 3 hours of chilling at -4°C. This was accompanied by a 45% (6 mg cm^-3^) decrease in starch levels. Assuming sugar levels increase primarily in living cells the observed increase in sugar concentrations may result in a freezing depression of 2°C [31]. This is sufficient to protect *P*. *integerrima* trees during most frosts in its natural habitat (mostly continental semi-arid environments) but would still fall short by a few nights each winter, implying a need for additional protection. In fact, the negative balance between SC accumulation and starch degradation during chilling of *P*. *integerrima* reveals irreversible carbohydrate use. A fraction of these carbohydrates were metabolized during respiration (ca. 10%) and the rest were potentially used as carbohydrate backbones to frost protection, e.g. to minimize dehydration accompanying intercellular ice formation [32]. *P*. *trichocarpa*, on the other hand, lacked excess respiration and did not alter its NSC levels as temperature dropped. This implies that *P*. *trichocarpa* may be less protected from frost-heat events or that it uses an alternative mode of protection that does not depend on carbohydrate metabolism. This evidence is only suggestive, however, and further inquiry is required to definitively link frost protection mechanisms to excess respiration.

The respirational increase observed in *P*. *integerrima* resulting from a single frost consumes only a small fraction of carbohydrate stored (ca. 0.01 mg cm^-3^ h^-1^). The additional SC observed in the cold is most likely synthesized back to starch as the tissue is reheated. Nevertheless, respiration induced by frost events remains elevated during warming until 9°C (Fig 2A) and the cycle repeats itself with each frost event (Fig 2B). In this case, repetitive excess respiration events could cumulatively deplete NSC storage over time, especially during periods of limited sugar supply (e.g. winter or drought). The effect would be amplified in tree compartments with high surface area and little storage (e.g. the canopy). As NSC deficiencies are associated with tree growth decline and mortality in severely water stressed ecosystems [2,4] the impact of long-term climate shifts on respiration and potential implications on tree vitality should be investigated further.

Annual climatic patterns will alter how strongly excess respiration depletes NSC supply. Consequently, the biological significance of excess respiration will vary spatially and temporally. According to our model, an average of 8.4 mg (glucose eq.) cm^-3^ yr^-1^ was consumed by trees due to cold-induced respiration in the western US from 2005 to 2013. In deciduous tree species, which experience winter dormancy, this level of NSC demand constitutes a substantial portion of available stem starch reserves. For example, in certain locations in California’s Central Valley where *P*. *integerrima* is cultivated we estimate that ~5 mg (glucose eq.) cm^-3^ yr^-1^ is lost to excess respiration with up to ~10 mg (glucose eq.) cm^-3^ yr^-1^ in more extreme years. Assuming initial starch levels like those measured in *P*. *integerrima* of ~12 mg (glucose eq.) cm^-3^ yr^-1^ (Fig 3B), depending on the year excess respiration could consume 42–83% of available starch reserves during winter dormancy (i.e. no photosynthetic activity). Excess respiration may also affect non-deciduous species during winter periods of limited carbon assimilation, e.g. during winter drought. Carbohydrate depletion of this magnitude may have significant impacts on spring vegetative growth. In the western US, average annual NSC demand due to excess respiration generally increases with elevation (Fig 4A). However, inter-annual variability in excess NSC demand is less associated with elevation (Fig 4B). Instead, it partly results from inter-annual temperature variability due to the influence of subarctic and subtropical gyres of the North Pacific, which are influenced by the strength and character of the California current system [33]. Our model, while highly simplified, highlights the potential significance of excess respiration with respect to carbohydrate depletion and points toward key areas of future research regarding the magnitude, timing, and location of this phenomenon.

Our novel finding that many trees increase respirational demand due to near-freezing temperatures reveals a potentially widespread, yet not previously reported risk to trees. This behavior is probably part of an adaptive mechanism which is deeply embedded in a species’ response to its habitat. It may also represent a small but persistent sink for stored carbohydrates that could meaningfully impact both natural and agricultural systems around the globe if climatic conditions change.

## Supporting Information

S1 Fig Percent increase from the projected stem respiration at -2°C in *P*. *integerrima* saplings and excised segments showed no significant differences between the methods (unpaired two-ways T-test, p>0.68, df = 9). (DOCX)

S1 Table Respirational response to a change in temperatures of 11 organisms presented in Fig 1A and their bibliographic references. (DOCX)
