# Supplementary material for: Frost Induces Respiration and Accelerates Carbon Depletion in Trees
Source: PLoS One. 2015 Dec 2;10(12):e0144124. doi: 10.1371/journal.pone.0144124 (PMC4668004; doi:10.1371/journal.pone.0144124)
Supplement: S1 Fig — (DOCX) [file pone.0144124.s001.docx]

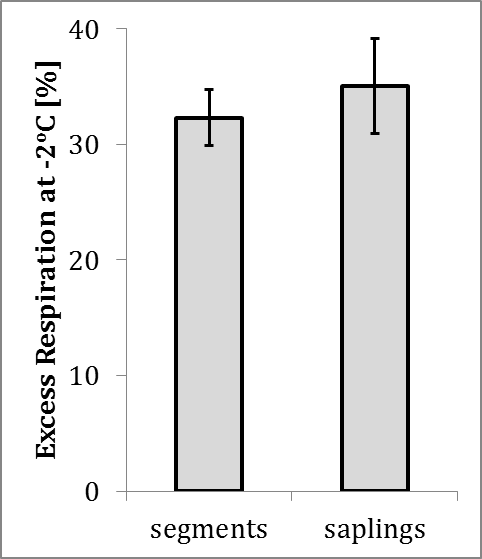


Fig. S1: Percent increase from the projected stem respiration at -2°C in *P. integerrima* saplings and excised segments showed no significant differences between the methods (unpaired two-ways T-test, p>0.68, df=9).
