# Supplementary material for: Frost Induces Respiration and Accelerates Carbon Depletion in Trees
Source: PLoS One. 2015 Dec 2;10(12):e0144124. doi: 10.1371/journal.pone.0144124 (PMC4668004; doi:10.1371/journal.pone.0144124)
Supplement: S1 Table — (DOCX) [file pone.0144124.s002.docx]

**References for figure 1A:**

| Ref | Organism | Q_10_ | Range [°C] |
| --- | --- | --- | --- |
| (1) | Humus soil | 2.3 | 0 to 40 |
| (2) | Forest soil | 2.7 | 2 to 15 |
| (3) | Grassland | 1.83 | 10 to 25 |
| (4) | *Pinus sylvestris* | 2.09 | -8 to 25 |
| (5) | *Abies balsamea* | 2.14 | 0 to 30 |
| (6) | *Gobius cobiti* | 2.3 | 12 to 25 |
| (7) | *Echis coloratus* | 1.92 | 10 to 35 |
| (8) | *Chrysemys picta bellii* | 2.32 | 10 to 30 |
| (9) | *Acer rubrum* | 1.7 | 3 to 20 |
| (10) | *Triticum aestivu* | 1.8 | 15 to 25 |
| (11) | *Saxifraga cernua* | 2.55 | 5 to 25 |

**Table S1:** Respirational response to a change in temperatures of 11 organisms presented in figure 1A and their bibliographic references:

1. Pietikäinen J, Pettersson M, Bååth E (2005) Comparison of temperature effects on soil respiration and bacterial and fungal growth rates. *FEMS Microbiol Ecol* 52:49–58.

2. Curiel yuste J, Janssens I a., Carrara A, Ceulemans R (2004) Annual Q10 of soil respiration reflects plant phenological patterns as well as temperature sensitivity. *Glob Chang Biol* 10:161–169.

3. Flanagan LB, Johnson BG (2005) Interacting effects of temperature, soil moisture and plant biomass production on ecosystem respiration in a northern temperate grassland. *Agric For Meteorol* 130:237–253.

4. Zha T, Kellomaeki S, Wang K (2004) Seasonal and annual stem respiration of Scots pine trees under boreal conditions. *Ann Bot* 94:889–96.

5. Lavigne MB, Franklin SE, Hunt Jr ER (1996) Estimating stem maintenance respiration rates of dissimilar balsam fir stands. *Tree Physiol* 16:687–95.

6. Berschick P (1987) The influence of hyperoxia, hypoxia and temperature on the respiratory physiology of the intertidal rockpool fish Gobius cobitis Pallas. *J Exp …* 387:369–387.

7. Al-Johany AM, Al-Sadoon MK (1996) Selected body temperature and metabolic rate–temperature curves of three species of desert snakes. *J Arid Environ* 34:363–370.

8. Glass M, Boutilier R, Heisler N (1985) Effects of body temperature on respiration, blood gases and acid-base status in the turtle Chrysemys picta bellii. *J Exp Biol* 51:37–51.

9. Edwards NT, Hanson PJ (1996) Stem respiration in a closed-canopy upland oak forest. *Tree Physiol* 16:433–439.

10. Gifford RM (1995) Whole plant respiration and photosynthesis of wheat under increased CO2 concentration and temperature: long-term vs. short-term distinctions for modelling. *Glob Chang Biol* 1:385–396.

11. Mcnulty AK, Cummins W (1987) The relationship between respiration and temperature in leaves of the arctic plant Saxifraga cernua. *Plant Cell Environ*:319–325.
